# Geographic variation in the damselfish-red alga cultivation mutualism in the Indo-West Pacific

**DOI:** 10.1186/1471-2148-10-185

**Published:** 2010-06-18

**Authors:** Hiroki Hata, Katsutoshi Watanabe, Makoto Kato

**Affiliations:** 1 Graduate School of Science, Kyoto University, Kitashirakawa-Oiwake, Sakyo, Kyoto 606-8502, Japan; 2 Graduate School of Human and Environmental Studies, Kyoto University, Yoshida-Nihonmatsu, Sakyo, Kyoto 606-8501, Japan; 3 Graduate School of Science and Engineering, Ehime University, 2-5 Bunkyo, Matsuyama, Ehime 790-8577, Japan

## Abstract

**Background:**

On coral reefs, damselfish defend their territories from invading herbivores and maintain algal turfs, from which they harvest filamentous algae. In southern Japan, intensive weeding of indigestible algae by *Stegastes nigricans *results in overgrowth by one filamentous alga, *Polysiphonia *sp. 1. Because this alga is highly susceptible to grazing and is competitively inferior to other algae, it survives only within the protective territories of this fish species, suggesting an obligate mutualism between damselfish and their cultivated alga. The wide distribution of damselfish species through the Indo-Central Pacific raises the question of whether this species-specific mutualism is maintained throughout the geographic range of the fish. To address this question, from all 18 damselfish species we conducted comprehensive surveys of algal flora within their territories throughout the Indo-West Pacific, and identified species of *Polysiphonia *using morphological examination and gene sequencing data.

**Results:**

Several species of the genus *Polysiphonia *were observed as a major crop in territories throughout the geographic range of *S. nigricans*. *Polysiphonia *sp. 1 occurred only in territories of *S. nigricans *in central areas of the Indo-Pacific. However, its occurrence was low from the Great Barrier Reef and Mauritius. In contrast, other indigenous *Polysiphonia *species, which formed a clade with *Polysiphonia *sp. 1, occurred in the territories of fishes from Egypt, Kenya, and the Maldives. The other *Polysiphonia *species in the clade only inhabited damselfish territories and were never found elsewhere.

**Conclusions:**

Cultivation mutualism between the damselfish *S. nigricans *and algae of *Polysiphonia *was maintained throughout the Indo-West Pacific, although algal crop species and the mode of cultivation (*e.g.*, presence/absence of selective weeding, the species composition of algal turfs) varied among localities. This finding implies that damselfish utilize indigenous *Polysiphonia *species in newly colonized habitats in different ways, and therefore the algal composition and means of cultivation have diverged.

## Background

Mutualism is widespread in nature, even between potentially antagonistic partners. Cultivation mutualism can be defined as relationships in which the consumer promotes the growth and net survival of the species it consumes. Most species engaging in cultivation mutualism are terrestrial, and these mutualisms usually involve fungi rather than primary producers (*e.g.*, ant-fungal mutualisms [1-3]). Mutualisms between primary producers and herbivores, with the exception of crops and humans, typically involve either protection of a few plants that are much larger than the herbivores (*e.g.*, ant-*Acacia *mutualisms [4,5]) or facilitate pollination (*e.g.*, fig-fig wasp mutualism [6,7]), and are thus not considered cultivation mutualism in the strict sense. Therefore, the cultivation mutualism between the territorial damselfish *Stegastes nigricans *and algae of *Polysiphonia *on coral reefs [8] is noteworthy by virtue of being marine and because it involves photosynthetic algae.

On coral reefs, damselfishes individually defend territories from invading vertebrate/invertebrate grazers and maintain algal turfs, from which they obtain all of their food [9,10]. These algal turfs are rich in biomass [11-14], are highly productive [12,15], and are dominated by delicate, filamentous rhodophyta, especially members of the genus *Polysiphonia *[14,16,17]. These damselfishes browse mainly on the upright axes of filamentous algae [18,19], digesting the material with a highly acidic stomach [20,21], and absorbing the digested material in their long intestine with a slow gut turnover rate [22-25]. They have neither masticatory organs nor effective endogenous or exogenous carbohydrases for breaking down algal cell walls, and thus can only digest filamentous algae that have no cortical layer [19-21,26,27]. The algal turfs of the territorial damselfish, *Stegastes nigricans*, are a monoculture of the filamentous red alga, *Polysiphonia *sp. 1, in Okinawa, Japan, which is the northernmost range of this species [14]. The fish intensively defends its territory against herbivores, weeds out indigestible corticated and/or calcareous algae, and harvests the filamentous alga. As a result, algal turfs within their small territories are dominated by only one algal species, *Polysiphonia *sp. 1, which is the most digestible alga species for this fish and is thus harvested by the damselfish as a staple food [19,28]. This *Polysiphonia *sp. 1 is susceptible to grazing by territorial invaders and from competition from other algae, and therefore, inhabits only the territories of this damselfish species where other herbivores are chased away and other algae are weeded out [8,29]. In this way, the fish and the alga are highly dependent on one another.

Reciprocal interactions between partners often vary geographically with the partners involved and the benefits received [30]. The alga-farming damselfish, *S. nigricans*, is widely distributed throughout the Indo-Central Pacific [31], and it is unclear whether its relationship with *Polysiphonia *species is maintained throughout its geographic range. This fish neither sows nor transplants the algae, and it is proposed that water-borne diaspores (carpospores and tetraspores) of the algae germinate into sporophytes/gametophytes [32,33]. However, little is known about the distribution and dispersal ability of *Polysiphonia *species. Damselfish larvae can potentially disperse over long distances, carried by currents during the pelagic larval stage, which lasts approximately 28 days [34,35]. However, genetic differentiation has been observed between populations of damselfish in the Maldives and Guam in Micronesia, suggesting that long-distance dispersal is rare [36]. The broad distribution of *S. nigricans *poses a question as to whether the cultivation mutualism occurring is also widespread. If so, is this partnership conserved throughout the geographical range of the fish? The composition of *S. nigricans *algal turfs have been reported from several sites covering a wide geographic distribution [14,16,37-39], however detailed and accurate classification of *Polysiphonia *to species level has thus far not been attempted. Morphological-based identification of filamentous red algae is highly problematic, mostly because they have a relatively simple morphology and exhibit extreme levels of phenotypic plasticity [40]. However, molecular identification using 18S rDNA sequencing is known to agree with the species taxonomy based on anatomical data in *Polysiphonia *algae [41]. The smallest difference between species was shown to be four bases in the 18S rDNA sequence [41], and as a result we used this criterion to distinguish species of *Polysiphonia *in this study. Thus, by determining the species identity of *Polysiphonia *algae using DNA sequencing data in concert with morphological characteristics, we explored the geographic pattern of the partnership between the fish and algal species, and detected the geographic distribution of the damselfish-algae mutualism.

## Results

**Figure 1:**
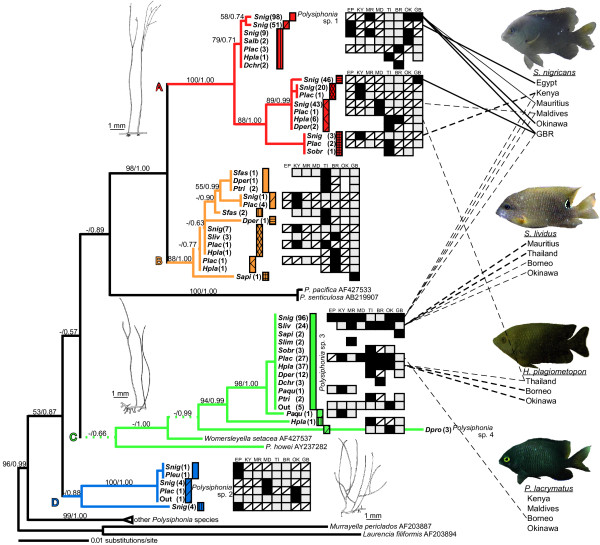
**Phylogeny of *Polysiphonia *algae found inside and outside the territories of 15 damselfish species**. Each alga is denoted by the abbreviation of its damselfish host species that is shown in Table 1. 'Out' indicates algae collected outside damselfish territories. Numbers in parentheses indicate the number of DNA samples including partially sequenced samples. Data presented as the scientific names of algae with accession numbers denote citations from the NCBI GenBank. Black blocks indicate the locations from which the algae were collected (EP, Egypt; KY, Kenya; MR, Mauritius; MD, the Maldives; TI, Thailand; BR, Borneo; OK, Okinawa; GB, the GBR). Gray blocks indicate areas inhabited by damselfish for which we have no data; blocks with slashes denote sites inhabited by damselfish where the alga was not found; open blocks indicate areas uninhabited by the fish. The tree was obtained using the ML method based on 1531 bp of 18S rDNA. Branches that collapse in MP, ML, and/or BI trees are presented as broken lines. Nodal support was assessed by the bootstrap value of MP and posterior probabilities of BI (above branches, MP/BI, respectively). Four abundant and widely-distributed damselfish species are listed on the right column, and their localities were linked with inhabitant *Polysiphonia *algae. Broad and narrow links indicate that the alga occurred in ≥ 50% or ≥ 25% of the territories of the damselfish species at these sites, respectively. Solid and broken lines indicate that the algal species occurred only within the territories of the fish species or that they also occurred in territories of other fish species and/or outside the damselfish territories, respectively.

We surveyed 320 territories of 18 damselfish species and thoroughly examined algae from each fish territory from coral reefs in Egypt, Kenya, Mauritius, the Maldives, Thailand, Borneo, the Okinawa Islands, and the Great Barrier Reef (GBR, Table 1). A total of 21 *Polysiphonia *genotypes were found among 211 territories of 15 damselfish species in the Indo-West Pacific (Fig. 1). These genotypes were different from each other by more than four bases in 18S rDNA, and therefore were treated as species. Molecular phylogenetic analyses of the collected *Polysiphonia *species detected four clades: A (including *Polysiphonia *sp. 1), B, C, and D, as shown in Fig. 1. *Polysiphonia *sp. 1, which had previously been found only within *Stegastes nigricans *territories in Okinawa, was discovered from territories of this damselfish species in Mauritius and the GBR, but nowhere else. *Polysiphonia *clade A species were seldom found outside territories of *S. nigricans*. In contrast, *Polysiphonia *sp. 3 of clade C occurred in the territories of 11 damselfish species and also outside damselfish territories throughout the study area. On the other hand, multiple species of *Polysiphonia *coexisted in the territories of *S. nigricans *and *Hemiglyphidodon plagiometopon *in some localities. In general *Polysiphonia *algae were rarely found outside damselfish territories. In Okinawa, two species of *Polysiphonia *clades C and D occurred in 9.5% and 0.6% of these extra-territorial samples, respectively, although a very closely related *Neosiphonia *species that lacks a prostrate axis occurred in 37.3% of the extra-territorial samples.

**Table 1:** Study sites and the number of samples collected.

Study site		Egypt	Kenya	Mauritius	Maldives	Thailand	Borneo	Okinawa	Great Barrier Reef
Latitude		N28°29'	S03°15' -S04°00'	S20°01'-27'	N4°10'-17'	N7°18'-24'	N5°58' -N6°03'	N24°18' -N26°42'	S19°10'
Longitude		E34°31'	E39°44' -E40°08'	E57°19'-48'	E73°29'-30'	E99°12'-17'	E116°00' -07'	E123°56' -E127°53'	E146°51'
*Stegastes nigricans*	(Snig)	10	10	23	11	0	0	40	23
*S. lividus*	(Sliv)	-	0	13	0	3	5	17	0
*S. albifasciatus*	(Salb)	-	0	-	0	0	0	1	0
*S. apicalis*	(Sapi)	-	-	-	-	-	-	-	8
*S. fasciolatus*	(Sfas)	-	0	0	0	3	0	0	0
*S. limbatus*	(Slim)	-	-	10	-	-	-	-	-
*S. obreptus*	(Sobr)	-	-	-	-	7	6	0	-
*S. pelicieri*	(Spel)	-	-	1	-	-	-	-	-
*Plectroglyphidodon lacrymatus*	(Plac)	4	15	0	8	6	12	13	0
*P. leucozonus*	(Pleu)	1	0	0	0	0	0	0	0
*Hemiglyphidodon plagiometopon*	(Hpla)	-	-	-	-	5	5	8	0
*Chrysiptera unimaculata*	(Cuni)	0	0	0	0	2	0	0	0
*Dischistodus chrysopoecilus*	(Dchr)	-	-	-	-	-	6	-	-
*D. perspicillatus*	(Dper)	-	-	-	-	6	2	-	0
*D. prosopotaenia*	(Dpro)	-	-	-	-	0	0	19	0
*Neoglyphidodon nigroris*	(Nnig)	-	-	-	-	2	0	0	0
*Pomacentrus aquilus*	(Paqu)	0	8	0	-	-	-	-	-
*P. tripunctatus*	(Ptri)	-	-	-	-	4	3	-	0
Outside territory	(Out)	0	0	0	0	0	0	158	0

- denotes the absence of the fish species at the site. Abbreviations of species in parentheses were used in Fig. 1.

**Figure 2:**
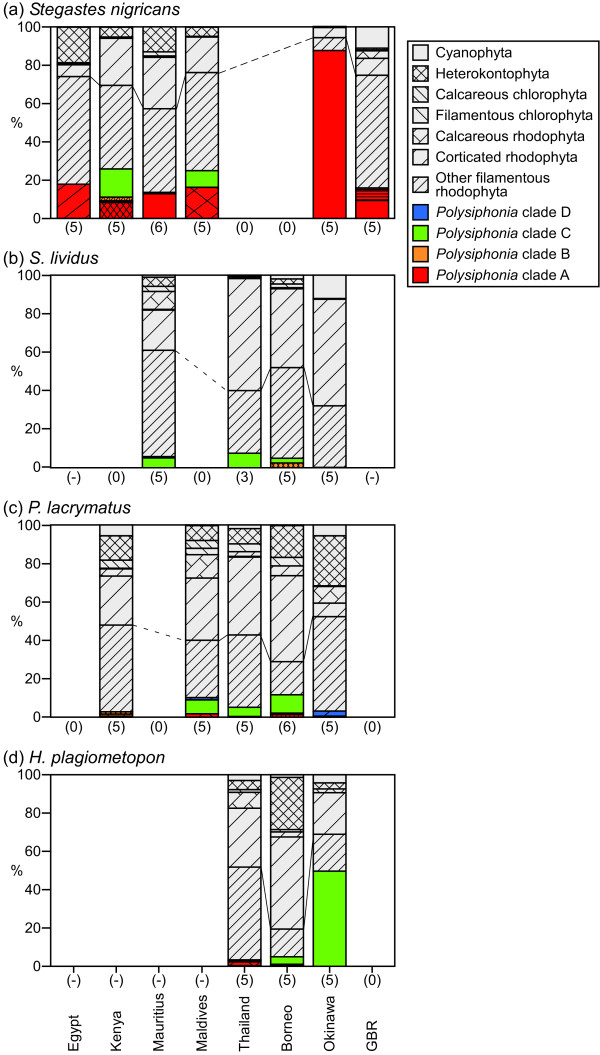
**Percent composition of algal turfs of four damselfish species in the Indo-West Pacific**. (a) *Stegastes nigricans*, (b) *S. lividus*, (c) *Plectroglyphidodon lacrymatus*, and (d) *Hemiglyphidodon plagiometopon*. Colors and patterns of areas correspond to clades and species of *Polysiphonia *shown in Fig. 1, *i.e.*, red, yellow, green, and blue indicate clades A, B, C, and D, respectively. Grey areas indicate composition of other algae. The numeral or hyphen (-) under each column indicates the number of samples or the absence of the fish species, respectively.

**Figure 3:**
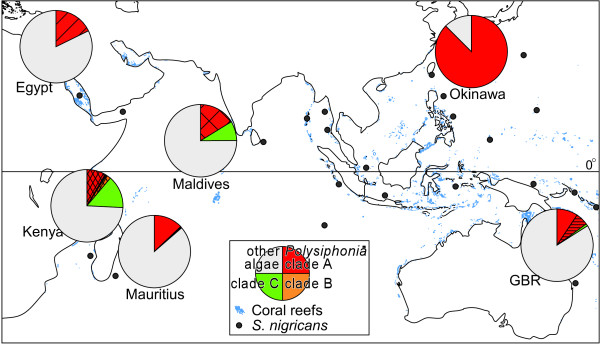
**Geographic mosaic of partnerships between *Stegastes nigricans *and *Polysiphonia *algae in the Indo-West Pacific**. The circle graphs indicate the biomass composition of algal species in territories of *S. nigricans *at our study sites. Colors and patterns correspond to the clades and species of *Polysiphonia *shown in Fig. 1, and grey indicates the presence of algae other than *Polysiphonia *spp. Light blue dots and black closed circles indicate the areas of coral reefs and *S. nigricans*, respectively.

Fig. 2 shows the percent composition of algae in the territories of four abundant and widely-distributed damselfish species, *S. nigricans, S. lividus, Plectroglyphidodon lacrymatus*, and *H. plagiometopon*. Algal compositions of the territories differed among fish species (two-way ANOSIM, R = 0.51, *p *= 0.001), and among study sites (two-way ANOSIM, R = 0.51, *p *= 0.001). Algal turfs of these damselfishes were dominated by filamentous rhodophyta including *Polysiphonia *algae. Instead of *Polysiphonia*, a mixture of filamentous algae of the genera *Neosiphonia*, *Herposiphonia*, *Anotrichium*, and *Ceramium *comprised substantial portions of the algal turfs in all damselfish territories from all localities except for territories of *S. nigricans *in Okinawa. However, *Polysiphonia *species in clade A, to which *Polysiphonia *sp. 1 belongs, were consistently the staple species in *S. nigricans *territories at all study sites, although occupancy varied among study sites. Occupancy was higher in Okinawa than at the other sites (Tukey-Kramer test, Okinawa vs. all other sites, all *p *< 0.05). *Polysiphonia *sp. 1 (plain red bar in Figs. 1, 2) dominated *S. nigricans *territories in Okinawa, and also occurred in territories in Mauritius and the GBR. However, coverage of *Polysiphonia *sp. 1 in territories of *S. nigricans *were significantly higher from Okinawa than from Mauritius or the GBR (Fig. 3; Tukey-Kramer test, both pairs, *p *< 0.05). In Okinawa, *S. nigricans *depended exclusively on this algal species, forming monocultures, whereas in Mauritius and the GBR, *Polysiphonia *sp. 1 did not always occur in the territories of *S. nigricans *and did not dominate where it did occur.

From Egypt, Kenya, the Maldives, and the GBR, other species of clade A occurred in the algal turfs of *S. nigricans*. The *Polysiphonia *species that was dominant in territories of *S. nigricans *from Egypt was a sister branch of *Polysiphonia *sp. 1 (Fig. 1). However, the dominant *Polysiphonia *species from Kenya and the Maldives were not closely related to *Polysiphonia *sp. 1, and they also inhabited the territories of other damselfish species. Other damselfishes, *i.e.*, *Stegastes lividus*, *Plectroglyphidodon lacrymatus*, and *H. plagiometopon*, shared *Polysiphonia *sp. 3 (plain green bar in Figs. 1, 2) in most localities.

## Discussion

### Mutualism and other relationships between territorial damselfishes and algae in the Indo-West Pacific

Throughout its geographical range, the territorial damselfish *Stegastes nigricans *maintained algal turfs that were consistently inhabited by local *Polysiphonia *species belonging to a single phylogenetic clade (clade A). *Polysiphonia *sp. 1 of clade A was common in the territories of *S. nigricans *from the central Indo-West Pacific, but not from Egypt, Kenya, or the Maldives, where other indigenous *Polysiphonia *species of clade A were utilized instead of *Polysiphonia *sp. 1 (Fig. 3). Two species of *Polysiphonia *that occurred in the territories of *S. nigricans *from Egypt and the GBR, and *Polysiphonia *sp. 1, were found only in *S. nigricans *territories. Additionally, two other *Polysiphonia *species were abundant and almost exclusively inhabited *S. nigricans *territories from the Maldives and Kenya. These results suggest that *S. nigricans *provides these *Polysiphonia *species with their sole or primary habitat, and harvests these filamentous algae as a staple food in all localities from the Indo-West Pacific. In this way, the mutualism between *S. nigricans *and its crop *Polysiphonia *species was maintained, but the partner *Polysiphonia *species varied among localities. Phylogenetic results suggest that shifts in crop algal species within clade A have occurred at least a few times in the West-Central Indian Ocean. Furthermore, the occurrence of *Polysiphonia *species in clade A in the territories of *S. nigricans *varied geographically, being highest in Okinawa, where the damselfish weed out algae other than the crop algal species. From Okinawa, where the relationship between the damselfish and *Polysiphonia *alga attains obligate mutualism, a single species of *Polysiphonia *dominated the territories of *S. nigricans *to form nearly pure monocultures, whereas other *Polysiphonia *species rarely coexisted within the territories. In the case of fungus-farming ants and termites, each colony is maintained as a monoculture of a genetically single cultivar [42,43], and this is thought to be a key stabilizing factor in the mutualistic interaction on an evolutionary time scale [44]. In the GBR and Mauritius, the coverage of *Polysiphonia *sp. 1 in territories was lower than in Japan, suggesting that the fish utilized not only this alga but also other algal species, as well as other resources such as detritus trapped in the algal turf [16,37].

Conversely, from most locations the algal turfs of three other species of damselfishes, *S. lividus*, *P. lacrymatus*, and *H. plagiometopon*, were dominated by filamentous rhodophyta, and shared a generalist *Polysiphonia *species. This discrepancy in the structure of algal turfs between *S. nigricans *and other damselfish species is caused by differences in the inherent farming strategies of fishes, that is, intensive farming and extensive farming. In the intensive farming method, damselfish defend their small monoculture patch by selective weeding and vigorous exclusion of invaders. In contrast, in extensive farming, damselfish defend large but mixed-species algal turfs only by excluding invaders [16,17,28,45].

The algal turfs of most damselfishes are known to be dominated by filamentous rhodophyta, but the dominant algal genera shift within the subclass Rhodymeniophycidae with geographic range as well as on much smaller scales such as reef zones on a coral reef [10,17], and species-specificity has been known only between a damselfish, *S. nigricans*, and *Polysiphonia *sp. 1 in Okinawa [8]. Extensive molecular phylogenetic analyses of algae enabled us to detect patterns between damselfish and *Polysiphonia *species at a species level, as this method is effective in detecting species interactions and coevolutionary processes in other mutualistic systems [46,47]. More intensive research on algal turfs of damselfishes at each location, and species identifications of *Polysiphonia *algae using DNA sequences are needed to understand the partnerships between damselfish and *Polysiphonia *algae, their degrees of specialization in this cultivation mutualism system, and their geographic variations. Other than our study sites and focus species, *Polysiphonia *species are known to be abundant in the territories of damselfishes all around the Pacific, including those of *S. apicalis *in the Gulf of Thailand [48], *S. lividus *in Guam [49], *S. nigricans*, *Neoglyphidodon nigroris*, *Plectroglyphidodon lacrymatus*, *and P. dickii *in Papua New Guinea [16,17], *Pomacentrus adelus*, *P. tripunctatus*, and *P. wardi *in the GBR [17], *S. nigricans *in Fiji [50], *S. fasciolatus *in Tonga [49], and *Microspathodon dorsalis *in the Gulf of California [51], and even in the Atlantic (*S. planifrons *in Jamaica) [52]. Studies of these species and locations would improve our understanding of mutualism between herbivorous fish and red algae on coral reefs.

### Geographic structure of the damselfish-red alga mutualism systems

The mutualism between a marine herbivorous fish, *S. nigricans*, and the filamentous rhodophyte *Polysiphonia *showed an asymmetric species-specific pattern in which indigenous *Polysiphonia *species exclusively inhabited the territories of *S. nigricans*, but not necessarily dominated the territories except in Okinawa. This mosaic pattern in the cultivation system is thought to be caused by differences in the history of interaction and coevolution between the fish and the algae and in the community of interacting organisms at each coral reef. Selective weeding by *S. nigricans *is a fundamental trait mediating this mutualism, and this maintains a near monoculture of *Polysiphonia *sp. 1 in Okinawa [8]. Weeding behavior by *S. nigricans *has been observed 0.5 times per 10 minutes in Okinawa [19], and 0.7 times per 10 minutes in Kenya (n = 3 individuals, 1 observation each, our primary observation). In Kenya, however, three species of *Polysiphonia *coexisted with each other and with other algae in the territories. On the other hand, weeding behavior was not observed from the Maldives at least in our observation (n = 5 individuals, 1 observation each, our primary observation) where territories of *S. nigricans *were inhabited by two species of *Polysiphonia*. Further study on the variation in weeding behavior, its efficiency in local conditions, and its genetic basis are needed to explain this discrepancy among localities. With respect to local environments, sea urchins were more abundant inside territories of *S. nigricans *in Okinawa than in Kenya and the Maldives, and in Okinawa invading herbivores were also much more abundant than in Kenya (Additional file 1: Fig. S1). Additionally, twelve species of territorial damselfishes coexist on reefs in Okinawa (Table 1). Therefore, the density, diversity, and composition of competitors may drive the selection mosaic [30]. Further studies on population genetics of damselfishes and *Polysiphonia *algae, and measurement of fitness under interactions in each locality in this geographic range will reveal how geographic variation is structured and whether the geographic mosaic theory of coevolution is valid [53].

Geographic variation in the partnership and specialization between the cultivation mutualism participants has also been reported between a fungus-growing termite and its crop fungus [54,55]. In this system, *Microtermes *termites are generalists and utilize genetically diverse symbiont fungi in South Africa, whereas the *Microtermes *species in western Africa (Senegal and Cameroon) are specialists and are associated with a single narrow lineage of fungi. The geographic patterns of mutualism are not obvious in fungus-growing attine ants or ambrosia beetles [56], but may be generally structured as observed from plant cultivation systems in humans [57].

## Conclusions

This study revealed that the intensive-farming damselfish *Stegastes nigricans *maintains similar algal turfs dominated or subdominated by a clade of *Polysiphonia *species throughout a wide geographic range, but that the crop alga species varies among distantly isolated localities. These *Polysiphonia *species inhabited territories of fish species exclusively or nearly exclusively. Therefore, cultivation mutualism between *S. nigricans *and *Polysiphonia *algae was maintained throughout the geographic range of *S. nigricans*, with some algal shifts in crops occurring from the West Indian Ocean and the Red Sea. This damselfish-alga mutualism is a model system through which we can approach the origin, establishment, and coevolutionary processes of the cultivation system.

## Methods

### Sampling

We collected *Polysiphonia *spp. and other algal species from the territories of 18 damselfish species from coral reefs at eight sites in the Indo-West Pacific from 2003 to 2006: Egypt (the Red Sea), Kenya, Mauritius, the Maldives, southern Thailand (the Andaman Sea), Sabah in Borneo, the Okinawan Islands in southern Japan, and the GBR in Australia (Table 1). Each algal sample was collected from each territory of damselfishes. We defined damselfish territory as the place where the territory holder fed on algae and defended against conspecific and heterospecific herbivores [58]. Whether a site was located within or outside of a damselfish territory was determined by 20 min of observation immediately prior to sampling. To facilitate thorough sampling of algae from outside the territories of fishes in Okinawa, Japan, we set line transects from the beach to offshore areas, perpendicular to the shoreline, at 2, 4, and 10 reef flats around Okinawa Island (N 26°04-52', E 127°38'-128°19'), Ishigaki Island (N 24°19-36', E 124°04-20'), and Iriomote Island (N 24°15-25', E 123°40-55') [6]. We set 1 × 1 m quadrats outside the territories at 50-m intervals along each transect and used a knife to scrape all of the algae and seagrass within the quadrats into mesh bags. In total, 158 samples were collected from outside damselfish territories.

Algal samples were immediately preserved in 100% ethanol. In the laboratory, samples were rinsed with distilled water, and all *Polysiphonia *algae were sorted under a microscope. *Polysiphonia *algae, including the small thalli of the algae, were classified into species using molecular data (see below). The percent composition of *Polysiphonia *species in algal turfs was quantified by spreading the algal samples evenly in a Petri dish marked with a grid, identifying the algae on fixed 100 crossing points using molecular data when necessary, and counting the occurrence of each algal species.

### Molecular methods

We extracted total DNA from field-collected, ethanol-preserved algae using Ampdirect^®^ Plus (Shimadzu, Japan). A fragment of the 18S ribosomal RNA gene was amplified by polymerase chain reaction (PCR) using the primers 5'-ACCTGGTTGATCCTGCCAG-3' and G07, and was directly sequenced using these two primers and four additional primers [59]. *Polysiphonia *species can be classified at the species level using 18S rDNA sequences [41]. All sequences were deposited in the DDBJ database [accession nos. AB505058-74].

### Phylogenetic analyses

Maximum parsimony (MP) and maximum likelihood (ML) analyses were conducted using PAUP* 4.0b10 [60], and Bayesian inference (BI) was conducted using MrBayes 3.2 [61]. MP analyses employed the heuristic search option with TBR branch-swapping and 1000 random-taxon-addition replicates, identifying the 40 most parsimonious trees of length = 447, C.I. = 0.593, and R.I. = 0.770. Heuristic MP bootstrap analysis consisted of 1000 pseudoreplicates with 10 random-taxon-addition replicates per pseudoreplicate. The likelihood ratio test implemented in ModelTest 3.06 [62] found the TrN + I + G model best fitting the sequence data, and this model was employed in a heuristic ML analysis. A heuristic search with 10 random-taxon-addition sequences and TBR branch swapping was performed. BI was performed based on the model HKY + I + G, which was selected by MrModeltest 2.3 [63], with 2,000,000 generations, sampling every 100 generations. The first 500 trees were discarded as burn-in after confirming chain stationarity from plots of likelihood against generation. The average standard deviation of split frequencies after 2,000,000 generations was 0.0033.

### Statistical analyses

Algal composition of damselfish territories were analyzed using two-way ANOSIM [64] with fish species and study site as factors. The percent composition of *Polysiphonia *species and that of *Polysiphonia *clades in the territories of *Stegastes nigricans *were compared among locations using a Tukey-Kramer test. ANOSIM was conducted using PRIMER 6 (Plymouth Marine Laboratories, UK).

## Authors' contributions

HH carried out the design of the study, field surveys, the molecular phylogenetic study, data analyses, and manuscript composition. KW carried out molecular phylogenetic study and manuscript composition. MK participated in the design of the study and carried out manuscript composition. All authors read and approved the final manuscript.

## Supplementary Material

Additional file 1 **Geographic variation in densities of herbivorous fishes and sea urchins inside territories of *Stegastes nigricans***. (a) frequencies in which *Stegastes nigricans *chased invading fishes out of their territories per 10 minutes, (b) the densities of sea urchins found inside the territories of *S. nigricans *in the three localities of Kenya, the Maldives, and Okinawa. Numbers in parentheses indicate the number of individual damselfish we observed. Different letters indicate significant differences at the 5% level by the Games-Howell test. *Observation data in Okinawa are cited from [28]. Click here for file
