# Supplementary figures and images for: Geographic variation in the damselfish-red alga cultivation mutualism in the Indo-West Pacific
Source: BMC Evol Biol. 2010 Jun 18;10:185. doi: 10.1186/1471-2148-10-185 (PMC2905425; doi:10.1186/1471-2148-10-185)

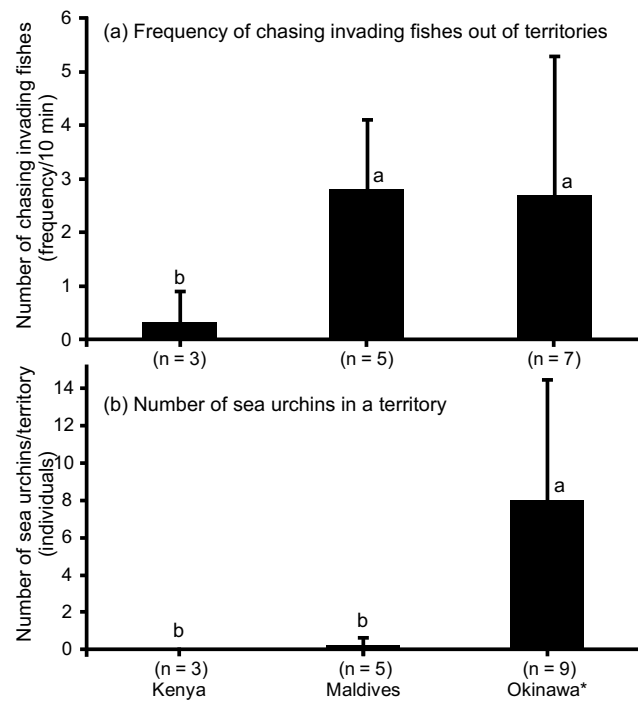

Supplement: Additional file 1 — Geographic variation in densities of herbivorous fishes and sea urchins inside territories of Stegastes nigricans. (a) frequencies in which Stegastes nigricans chased invading fishes out of their territories per 10 minutes, (b) the densities of sea urchins found inside the territories of S. nigricans in the three localities of Kenya, the Maldives, and Okinawa. Numbers in parentheses indicate the number of individual damselfish we observed. Different letters indicate significant differences at the 5% level by the Games-Howell test. *Observation data in Okinawa are cited from [28]. [file 1471-2148-10-185-S1.PDF]
